# The Medical Curriculum

**Published:** 1899-09-02

**Authors:** 


### The Medical Curriculum.

With many people, and indeed with most of those who are engaged in the work of education, September comes as an interlude between work and play. At the commencement of the long vacation, what with the weather, the fag which comes from the grind of previous months, the craving for change, and the longing for rest at any price, every one gladly casts work and even the thought of work aside. But we are creatures of habit. Even holidays soon pall upon those whose lives are mostly spent in work, and with the cooler air, and the sense of refreshment given by travel and change of scene, this month finds us waking up, and not altogether sorrowfully, to the fact that soon we shall be once more in October, and shall find ourselves again surrounded by the rush and grind of that student life of which every great hospital is a centre. To parents and guardians, and to the lads themselves who are about to enter on a medical career, September is a month of much anxiety, for then at last the great question, " Where to go," must be definitely decided, a medical school must be chosen, and all arrangements must be finally made, for in October actual work must be begun.

The responsibility entailed in this decision is made all the greater by the extraordinary ignorance which seems to prevail among many people not only as to the prospects offered by a medical career, but as to the training and education which are necessary before a qualification can be obtained. The inquiries which we are constantly receiving on these subjects tend not only to show how little some people who are about to enter the medical profession know what is before them, but to suggest that in many cases the whole subject has become surrounded in their minds with a halo of mystery and romance which is totally unwarranted by the prosaic details of a medical education and the disagreeable and sometimes disgusting surroundings amid which much medical work has to be done. We think, then, that a little accurate information in regard to the course of study to be pursued will be acceptable to those who are proposing to enter upon the study of medicine, and to them, rather than to our regular readers, we therefore address the following remarks.

### The "Conjoint."

Whatever may be the case in the future there can be no doubt that for some time to come the diplomas granted by the Royal Colleges of Physicians and of Surgeons will remain the standard qualifications which will be taken by the great mass of the best medical students. We unhesitatingly advise all who can do so to obtain a degree from a university. In many ways and for many reasons such a distinction is very useful, but this in no way detracts from the importance of obtaining the conjoint qualification, for it has to be remembered that the highest posts in connection with the London hospitals are open only to those who possess, on the medical side, the membership of the College of Physicians, and on the surgical side the fellowship of the College of Surgeons. Hence the advantage of diplomas, which, although obtained after a single series of examinations, are stepping stones to the higher qualifications of each body. In the following description of the course of study which has to be undertaken by the student of medicine we have then supposed that he is aiming at the M.R.C.S., L.R.C.P., but as a fact there is but very little difference in the general arrangement of the course whatever qualification is to be taken, except that for those aiming at the degree of the University of London no lectures on anatomy and physiology will count until the preliminary scientific examination has been passed, and that those entering at the other universities must take care that the studies and the examinations follow each other in due order, and that certain conditions as to residence are complied with, all of which, of course, must be ascertained from the authorities connected with the university in question.

### The Preliminary Examination.

The first thing that the would-be medical student has to do is to register as such—for the obligatory five years' course of study is always counted from the date of registration. For this purpose application must be made to the Registrar of the General Medical Council, 299, Oxford Street, London, W. The student, however, cannot be registered until he has on the one hand (*a*) passed a preliminary examination in arts recognised by the General Medical Council, and on the other has (*b*) commenced medical study. In regard to the preliminary examination, this must embrace the following subjects, which must all be passed at one and the same time:—

(*a*) English language, with grammar and composition (marks not exceeding 5 per cent. of the total marks obtainable in this section of the examination may be assigned to candidates who show a competent knowledge of shorthand).

(*b*) Latin, including grammar, translation from set authors, and translation of easy unseen passages.

(*c*) Mathematics, comprising (1) arithmetic; (2) algebra (as far as simple equations inclusive); (3) geometry (Euclid, books I.-III.)

(*d*) One optional subject: (1) Greek, or (2) French, or (3) German, or (4) any other modern language.

A complete list of the recognised examinations may be obtained on application to the Registrar of the General Medical Council.

If the student has not already passed any of the recognised examinations mentioned in the above-mentioned list, one or other of the following examinations will probably be found the most suitable for the purpose: (1) The Matriculation of the University of London; (2) the Examination of the College of Preceptors; (3) the Junior Local Examination of the Universities of Oxford or Cambridge; taking care that these include all the necessary subjects, as given above. Of these examinations the matriculation of the University of London is the most difficult, but it is by far the best, because if the student should find that he has both time and brains enough to enable him to take his degree at the London University, he can, after passing this examination, go on without delay; for it must be understood that if the student is to obtain the degree of M.B. London he must pass the matriculation examination of that University.

The following addresses may be found useful by those who desire further information in regard to these preliminary examinations :—

*University of London*—To the Registrar of the University of London, Burlington Gardens, London, W.

*College of Preceptors*—To the Secretary, College of Preceptors, Bloomsbury Square, W.C.

*Cambridge Junior Local*—To the Secretary of the Local Examinations, Syndicate Buildings, Mill Lane, Cambridge.

*Oxford Junior Local*—To the Secretary, Local Examination Offices, Merton Street, Oxford.

### Precocious Boys.

Some clever and precocious boys are able to pass the preliminary quite early, and then it becomes a question what to do with them. Under recent regulations it is possible for them to pass the first of the five years of medical study at certain "recognised teaching institutions" other than universities or medical schools; this year being spent in the study of chemistry, physics, practical chemistry, and elementary biology. Instruction in practical pharmacy may also be taken in this year away from a medical school, and indeed may be taken before registration. Thus it is possible to get rid of the whole of the subjects for the first examination before joining a real medical school. There is no doubt that for a boy who is able to pass his preliminary examination early, and who happens to be studying at one of the schools which are recognised teaching institutions, there are certain advantages in this course. For one thing he is kept under the master's eye for another year, which is an important consideration when a lad happens to be able to pass the preliminary at 15 or 16 years of age, and another advantage is that a year at school, especially if it be a day school, costs much less money than a year spent in a great town as a student of medicine. But we very much question whether it will ever be desirable for a student who, as a school boy, has not been connected with one of these recognised teaching institutions, to enter at such a place for his first year. He had much better go at once to his medical school There is no doubt, we think, that it is always a disadvantage to enter a medical school in one's second year, when all the fellows have shaken down into their "sets," and although this may be faced for the sake of staying on for another year at one's own old school, it certainly is not worth while risking it under other circumstances.

The question whether it is worth while passing the preliminary at so early an age at all is one which depends entirely upon monetary considerations. If it is desirable to qualify, and to get into practice at as early an age as possible, there is no doubt that the sooner one is registered the better. But if a good position is aimed at rather than an early qualification, we would say stay at school, or even go to a university and take an arts degree; in any case, get as good a general educaticn as possible before touching medicine at all.

### The Commencement of Medical Study.

To "enter" at a medical school is the simplest matter in the world to one who has passed one or other of the prescribed examinations and possesses the necessary money. All he has to do is to attend at the office, produce his certificate of having passed his preliminary, together generally with a certificate of character, and pay the entrance fee. He then signs the register of students, promises to obey the regulations, and receives a certificate of having commenced medical study. Within fourteen days of "entering" he should register his name at the office of the General Medical Council, and then he becomes a registered medical student, and all the work that he does and all the lectures which he attends "count"—that is, are recognised as part of the prescribed course which he has to go through between registering as a medical student and qualifying as a medical man or woman, as the case may be, not less than five years later.

Except for the novelty of the thing, and the fact that the subjects of study are quite different from all that he has hitherto been accustomed to, the work of the first two years is apt to be found a trifle dull. To some, in fact, it would be not only dull, but loathsome, were it not that at a good medical school everything is arranged in steps and stages each of which leads to an examination, so that the element of competition is introduced from the very beginning. We strongly advise the student to enter with spirit from the very commencement into all these class examinations. We often hear it said that knowledge should be sought for its own sake, which is all very fine, but we doubt whether that is a sentiment which will spur many young fellows on to the making of elaborate dissections of trumpery superficial nerves, and go to all the apparently aimless labour which is necessary if they are to get a good working knowledge of their anatomy. But youth is always competitive, and if students would give as full play to the spirit of competition even in those much maligned things "class examinations" as they do in their sports they will find that even the grubby details of dissecting become full of interest.

### First Winter Session.

To begin then with the work of the first winter session. In this the student will attend lectures on elementary anatomy, biology, and chemistry, and will do practical work in each subject. In all these subjects steady work must be done, and the student will find that he has his hands pretty full if he is to keep pace in his reading with the lectures and demonstrations. Biology must especially be attended to, because if he wishes to avoid losing time—and loss of time always means increased expense—he must be able at the end of his first winter to pass the biological part of the first examination. If, however, he accomplishes this satisfactorily he then during the

### First Summer Session studies chemistry, physics, and physiology, and also materia medica and practical pharmacy. At the end of this session he should be able to pass the chemistry and physics parts of the first examination, and, if possible, the examination on practical pharmacy.

### Second Winter Session.

He thus gets entirely rid of the elementary subjects, and in his second winter session he is able to devote himself exclusively to anatomy and physiology, which are the subjects on which he will next be examined.

### Second Summer Session.

In the second summer session, however, when he cannot well be dissecting, he will at some schools begin to get a little clinical work in the hospital.

At some schools it is the rule that no hospital work is to be done at all until the second examination has been passed, but in others students are recommended to attend medical and surgical practice during the second summer session, and to take out certain preparatory classes for dressers and clinical clerks. If a student enters in May the latter may be a very good plan, because he goes up for his second examination at the end of a winter session, and thus is fresh from the work on which he is to be examined. But if he enters in October, and thus has to take his second examination at the end of a summer session, we greatly doubt the advantage of entering upon a lot of fresh subjects just before going in. Practically it is found that students who desire to pass the second examination at the end of the summer session have their work pretty well cut out for them without taking up new subjects.

### Third Winter and Following Sessions.

Supposing, then, this examination is passed—and unfortunately it is an examination in which failures occur very frequently—the student in his third winter session finds himself launched into a new world. At last he is in the hospital, studying practice instead of theory, studying humanity instead of bones. Every student who has in him the making of a good practitioner feels the joy of getting into the wards. Now at last he is in touch with his life's work; and, moreover, there are no more examinations for a long time to come, which is always felt as a great relief.

During the following two years the student will find his time fully occupied in going systematically through the various appointments open to him in the hospital. Medicine, surgery, and pathology will be studied in the wards, in the operating theatre, and in the dead-house; midwifery will be practically worked at in the maternity department, and lectures on all sorts of subjects will be attended, and altogether the student will feel that he never was so busy in his life. At the end of this time he will, if he is wise, pass the midwifery portion of his final examination.

### The Fifth Year.

The fifth year may be devoted to the study of any of the specialities which may happen to have been omitted, to the study of fevers at a fever hospital, to further clinical work, to attendance at a lunatic asylum, to vaccination, and to various other duties, among which must be reckoned attendance on special tutorial classes for the final examination, and then at the end of this fifth year the final may be passed.

### The Importance of Punctuality in Passing the Examinations.

From every point of view, and especially from that of the parent who has to provide the money, it is impossible to exaggerate the importance of sticking strictly to the curriculum and passing the examinations at the appointed times. It will be noted when the details come to be investigated that certain courses must be taken between certain examinations, and thus it happens that delay in passing an examination may hamper one all along. Even if it do not actually involve lengthening the time to be spent at the medical school, which, of course, greatly increases the expense, a delay in one of the earlier examinations may so dislocate the course that the examinations do not follow the appropriate studies as they ought to do, and that the work is thus so complicated as to render the examinations distinctly harder to pass, so that on all accounts it is worth much effort to pass punctually. So important, in fact, is it from a cash point of view, as well as from that of mere kudos, that it often pays well to have a grinder for a subject on which one may feel any doubt. Anything rather than be left behind and thrown among a set of men junior to oneself.

Of late years the examinations have been so severe that in estimating the expense of a medical education we would not advise a parent to cut it too fine, or to count too confidently on the curriculum being gone through without a "plough" on one or other of the subjects.

